# 16306 Genomic epidemiology of SARS-CoV-2 across New Mexico and the Mountain West

**DOI:** 10.1017/cts.2021.696

**Published:** 2021-03-30

**Authors:** Daryl Domman, Kurt Schwalm, Twila Kunde, Joseph Hicks, Michael Edwards, Noah Hull, Wanda Manley, Ethan Romero-Severson, Emma Goldberg, Darrell Dinwiddie

**Affiliations:** 1 University of New Mexico Health Sciences Center; 2 New Mexico Department of Health; 3 Wyoming Public Health Laboratory; 4 Los Alamos National Laboratory

## Abstract

ABSTRACT IMPACT: Genomic data can be used by policy and decision makers to guide, and assess the impact of, public health responses to the COVID-19 pandemic. OBJECTIVES/GOALS: Our objective is to investigate the transmission and population dynamics of SARS-CoV-2 in New Mexico and other Mountain West states using whole genome sequencing. Understanding how the virus is spreading within and between communities is vital for the design of rational, evidence-based control measures. METHODS/STUDY POPULATION: We obtained an aliquot of 500ul - 1 ml of inactivated viral transport media (VTM) from positive SARS-CoV-2 nasopharyngeal swabs as determined by qPCR from the New Mexico Department of Health, TriCore Reference Laboratory, Idaho Bureau of Laboratories, and Wyoming Public Health Laboratory. We extracted viral RNA from the VTM, and sequenced the genomes using the methodology as described by the widely adopted ARTIC amplicon tiling protocol for SARS-CoV-2. Viral genomes were then sequenced on either an Illumina MiSeq or an Oxford Nanopore Technologies (ONT) GridION. We placed these samples within the context of globally representative sequences made available via the GISAID database. Consensus sequences were aligned and added into this global dataset using the Nextstrain augur pipeline. RESULTS/ANTICIPATED RESULTS: We sequenced over 1,000 SARS-CoV-2 genomes thus far from New Mexico (n=861), Wyoming (n=213) and Idaho (n=44). We used this sequence data to infer the transmission dynamics and spread of the virus, both within states and in context of regional and international spread. We inferred at least 128 separate introductions of the virus into New Mexico and at least 29 introductions into Wyoming. The origins of these introductions are diverse, spread across multiple regions in the US and abroad. We also sequenced samples from an individual who had multiple positive tests over time. Our results suggest that this individual was re-infected with a different strain than that of the initial infection. DISCUSSION/SIGNIFICANCE OF FINDINGS: Our data show that New Mexico and other Mountain West states have continually experienced many introductions of the virus that then seed local outbreaks. By understanding the number of introductions over time, we can assess the impact of travel restrictions on transmission. Our data also supports that some individuals can be re-infected.

